# Protamine expression in somatic cells condenses chromatin and disrupts transcription without altering DNA methylation

**DOI:** 10.1186/s13072-025-00633-2

**Published:** 2025-10-04

**Authors:** Deepika Puri, Alexandra Bott, Monica Varona Baranda, Esra Dursun Torlak, Gina Esther Merges, Hubert Schorle, Wolfgang Wagner

**Affiliations:** 1 Institute of Stem Cell Biology, University Hospital of RWTH Aachen, 52074 Aachen, Germany; 2 Helmholtz-Institute for Biomedical Engineering, Medical Faculty of RWTH Aachen University, 52074 Aachen, Germany; 3 Department of Developmental Pathology, Institute of Pathology, University Hospital Bonn, 53127 Bonn, Germany

**Keywords:** Protamine, DNA methylation, Nuclear condensation, PRM1, PRM2, Transcription

## Abstract

**Background:**

Protamines play a crucial role in nuclear condensation during spermiogenesis, a process associated with significant chromatin remodeling and replacement of histones. While much research has focused on the function of protamines in sperm development and fertility, their effects in non-sperm cells remain largely unexplored. Protamines are increasingly used in the clinical setting, and understanding better, the role of protamines in somatic cells remains a critical unmet need.

**Results:**

In this study, we investigated the impact of overexpressing murine and human protamine 1 and 2 (PRM1 and PRM2) on nuclear architecture, histone eviction, DNA methylation, and transcription in HEK293T cells and mesenchymal stromal cells (MSCs). Overexpression of protamines resulted in nuclear condensation; particularly PRM1 showed notable enrichment in nucleoli, and cells exhibited cell cycle abnormalities. Immunofluorescence staining indicated a significant reduction in specific histone modifications (H3K9me3, H3K4me1, and H3K27Ac) in response to protamine expression, especially in MSCs. Interestingly, despite these changes in nuclear organization, the methylome remained largely stable. However, expression of protamines significantly diminished transcription, particularly of the ribosomal genes, upon PRM1 expression.

**Conclusions:**

Our studies indicate that PRM1 and PRM2 may bind to and condense distinct genomic regions in somatic cells, resulting in widespread silencing of gene expression, while retaining a largely stable DNA methylome.

**Supplementary Information:**

The online version contains supplementary material available at 10.1186/s13072-025-00633-2.

## Introduction

Protamines are nucleoproteins that facilitate the higher-order compaction of sperm chromatin to protect the genetic integrity of the paternal genome [1–3]. Spermatids of all mammals express protamine 1 (PRM1), whereas rodents and primates additionally express protamine 2 (PRM2) [1], with their expression ratio being important for fertility [2]. Protamines participate in a cascade of events during spermiogenesis that result in histone replacement, a sharp decline in transcription, and nuclear condensation [2]. The chromatin structure is severely altered by the binding of protamines, predominantly to the major groove, neutralizing the phosphodiester backbone charge [1]. *PRM2* is transcribed as a precursor; the N-terminal region of the translated PRM2 is cleaved during spermiogenesis, and the mature PRM2 associates with the condensing DNA [4]. During sperm development, protamines replace most histones, and this histone-to-protamine transition involves the replacement of somatic histones with testis-specific variants and the incorporation of transition proteins, followed by protamines [5, 6]. Notably, a portion of histones and their modifications are retained and are thought to contribute to transmitting epigenetic memory from the sperm to the embryo [7, 8].

The histone code is intertwined with the DNA methylation pattern [9, 10], and there is crosstalk between the two in development as well as diseases [11, 12]. Therefore, the histone-protamine transition may alter the methylome, but this has so far not been investigated. Analysis of global DNA methylation levels with either enzyme-linked immunosorbent assay or immunofluorescence analysis of 5-methylcytosine suggested that protamine deficiency in sperm is associated with increased global DNA methylation [13, 14]. Furthermore, sperm from patients with protamine deficiency showed higher DNA methylation at six of seven tested imprinted control genes [15], but it remains unclear if protamines contribute directly to this change in DNA methylation. Additionally, as protamination leads to condensation of sperm DNA up to six times more than during a mitotic cycle, access to the transcriptional machinery is severely impaired, resulting in near-complete silencing of transcription in sperm [16].

While the nuclear compaction during spermiogenesis has been well studied, there is little understanding of the effect of protamine expression in somatic cells. Previous studies have shown partial condensation of sheep fibroblast nuclei upon overexpression of hPRM1; condensation of HEK293T nuclei upon overexpression of mPRM2, and reduced proliferation of HeLa cells upon protamine overexpression [17–19]. Investigating the role of protamines in somatic cells is crucial, especially as protamines are increasingly used as adjuvants for mRNA vaccines with applications in nanopharmaceuticals [20, 21], and as stabilizers for transfection [22]. To bridge this gap, we have comprehensively analyzed the effect of overexpressing human or murine PRM1 and PRM2 in different cell types. Our results shed light on the effect of protamine overexpression on transcription, histone modifications, and DNA methylation in somatic cells and emphasize the distinct function of these proteins in chromatin condensation.

## Experimental procedures

### Cell culture

The HEK293T cells were cultured in Dulbecco’s Modified Eagle Medium (DMEM), high glucose, supplemented with 10% fetal bovine serum (FBS) and 1% penicillin-streptomycin at 37 °C in a humidified atmosphere with 5% CO2. MSCs were isolated from human bone-marrow samples of patients undergoing orthopaedic surgery after written and informed consent (ethics approval EK300/13). The cells were cultured in DMEM, low-glucose, supplemented with pooled human platelet lysate (10%), L-glutamine (2 mM), penicillin–streptomycin (100 U ml^− 1^) and heparin (5 IU ml^− 1^) at 37 °C and 5% CO2. Mouse embryonic fibroblasts (MEFs) were prepared from WT 129s2/Sv mice as described before [1], and cultured in DMEM containing Glutamax, 4.5 g/l D-Glucose, and Pyruvate, 10% FBS, 1x essential amino acids, 1x non-essential amino acids, 1x L-Glutamine, 50 µg/ml Penicillin/Streptomycin. The cell culture media were changed every 2–3 days, and the cells were passaged when the monolayer reached a confluency of 80–90%.

### Plasmids and transfection of cells

The plasmids pcDNA3.1-EGFP, pcDNA3.1-EGFP-hPRM1 and pcDNA-3.1-EGFP-hPRM2 were purchased from Genescript. The pEGFP-N3-mPrm2 plasmid was generated as described before [2]. pEGFP-N3-mPrm1 plasmid was constructed using the pEGFP_N3 (Clontech (#6080-1)) plasmid and *Prm1* amplified from C57Bl/6J mouse testis cDNA in the same manner and provided by the Schorle lab. The following overhang primers were used: Prm1_EcoRI_fwd (AAAAG AATTC ATGGC CAGAT ACCGAT G); Prm1_BamHI_rev (TTTTG GATCC GTATTT TTTAC ACCTT ATGGT G). Both human and mouse protamine 2 constructs express full length transcripts.

HEK293T cells were transfected with the plasmids, using the TransIT-LT1 transfection reagent (Mirus Bio). Three days post-transfection, HEK293T cells were sorted, and live, high EGFP-positive cells were used for further experiments. MSCs were transfected by electroporation using the NEON Transfection System using optimized protocols. 24 h post-transfection, cells were selected with geneticin at 800 µg/ml for 4 days. MEFs after passage 3 were transfected on 6-well plates at a confluency of approximately 50% in antibiotic-free MEF medium. The FUGENE HD Transfection reagent (Promega) was used according to the manufacturer’s instructions. Each well was transfected with 4 µg of plasmid. Cells were harvested 48 h after transfection.

### Antibody staining and nuclear area measurement

Cells were fixed with 4% paraformaldehyde (PFA) for 20 min and permeabilized with PBS containing 1% w/v BSA and 0.1% v/v Triton X-100 (Bio-Rad) for 30 min, followed by overnight incubation at 4 °C with primary antibodies, as mentioned in (Supplemental Table 2). Secondary antibody (Supplemental Table 2) staining was done at room temperature (RT) for 1 h. Samples were then counterstained with DAPI (10 ng/mL) for 15 min at RT in the dark. The coverslips were then mounted on glass slides using mounting medium (DAKO) and samples were imaged using a Zeiss Axio Observer fluorescence microscope using 40x or 63x objective as described before [23]. FIJI [24] was used for the quantification of the nuclear area. Violin plots visualized the distribution of measured nuclear area values, and statistical significance was tested using the Mann-Whitney U test. The intensity of the histone modification staining was measured using FIJI using the mean integrated density measurements of the region of interest (ROI) identified based on DAPI staining. Statistical significance between conditions was tested using the Mann-Whitney U test. For imaging of mitotic chromosomes, HEK293T cells were seeded on coverslips 24 h post transfection, without sorting, grown overnight and stained with DAPI the next day.

### Quantitative reverse transcription polymerase chain reaction (qRT-PCR)

Total RNA was isolated from HEK293T cells using the NucleoSpin RNA Plus Kit (Macherey-Nagel), quantified with a NanoDrop ND-2000 spectrophotometer (Thermo Scientific), and converted into cDNA using the High-Capacity cDNA Reverse Transcription Kit (Applied Biosystems). The cDNA was amplified using Semi-quantitative reverse-transcriptase PCR (RT-qPCR) using Power SYBR Green PCR Master Mix (Applied Biosystems) in a StepOnePlus machine (Applied Biosystems, Waltham) using primers described in (Supplemental Table 1) to target the human *PRM1* and *PRM2* genes and the mouse *Prm1* and *Prm2* genes. *GAPDH* was used as an internal control. The relative expression of the protamine samples was normalized to the relative expression of the empty vector samples to display the fold change.

### Apoptosis assay

Three days after transfection, HEK293T cells were collected and stained with Alexa Fluor 647 Annexin V solution and 5 µl of a 20 µg/ml DAPI solution following standard protocols and analyzed with the BD FACS Canto II. Stacked bar plots were generated to visualize the distribution across the four categories, and statistical significance between conditions was tested using Welch’s t-test.

### Cell cycle analysis

Three days after transfection, approximately 500,000 HEK293T cells per condition were stained with 1 µg/ml DAPI solution and transferred to FACS tubes. After 15 min of incubation, the cells were analyzed by flow cytometry with the BD FACS Canto II. Gates for the cell cycle phases were set on the DAPI histogram for each population. The cell cycle phases were analyzed in the high EGFP-positive population. Stacked bar plots were generated to visualize the distribution across the cell cycle phases, and statistical significance between conditions was tested using Welch’s t-test.

### DNA methylation analysis

DNA methylation profiles were analyzed in HEK293T cells transfected with empty vector, hPRM1, or hPRM2 using the Illumina Methylation EPIC v2.0 BeadChip. DNA from three replicate samples for each condition was isolated using the NucleoSpin Tissue kit (Macherey Nagel), and microarray analysis was performed at Life and Brain (Bonn, Germany). The R packages minfi (v.1.48.0) [25], SeSAMe (V 1.20.0) [26], limma [27], and missMethyl [28] were used for data import, preprocessing, quality control, and analysis, respectively. A multidimensional scaling (MDS) plot was created to visualize the overall similarity of methylation profiles across the different samples for the top 10,000 most variable CpG sites. Scatterplots of the mean beta values of the two conditions were created to visualize differentially methylated CpG sites (at least 20% difference of mean methylation).

### Transcriptomic analysis

Total RNA from HEK293T cells transfected with empty vector, hPRM1 and hPRM2, was isolated using the NucleoSpin RNA Plus kit. mRNA sequencing (mRNA-seq) with 3′-end enrichment was then performed using the QuantSeq 3′ mRNA Library Prep Kit (Lexogen) and sequenced on a NovaSeq 6000 platform. The mRNA-seq data was processed using the nf-core/rnaseq pipeline [29]. The pipeline involved quality control with FastQC, adapter trimming using Cutadapt, pseudoalignment of reads to the annotated reference genome using STAR [30], and production of a gene expression matrix using Salmon [31]. Further normalization and analysis of the data was performed in R. Briefly, expression matrices were uploaded to R and mitochondrial genes were removed. Normalization is a crucial step to identify biologically meaningful differentially expressed genes. For this purpose, DESeq [32] is used, that assumes equal read counts to determine differential expression based on negative binomial distribution. Since there is a disbalance in the number of total reads obtained from the protamine expressing samples, compared to the empty vector, normalization was performed by total number of reads per sample to obtain biologically meaningful results, as described in [33, 34]. In short, we opted to normalize the samples by library size, computing reads per kilo base of transcript per million mapped reads (RPKM) for each transcript. To obtain the RPKM of each gene, the read count of each gene was divided by the total count of reads per sample divided by a million, obtaining the RPM of reads per million that serves as a normalization for sequencing depth. Finally, RPM is divided by the length of the gene, in kilobases, obtaining RPKM. Differentially expressed genes were computed using a t-test with p-values ≤ 0.05 adjusted by FDR. The “Counts Biotypes” tool was used to classify and quantify the reads based on the gene biotype annotations (e.g. protein-coding, pseudogene, lincRNA, etc.), by counting the number of reads that overlap with annotated gene features.

## Results

### Protamine expression leads to nuclear condensation

**Fig. 1:**
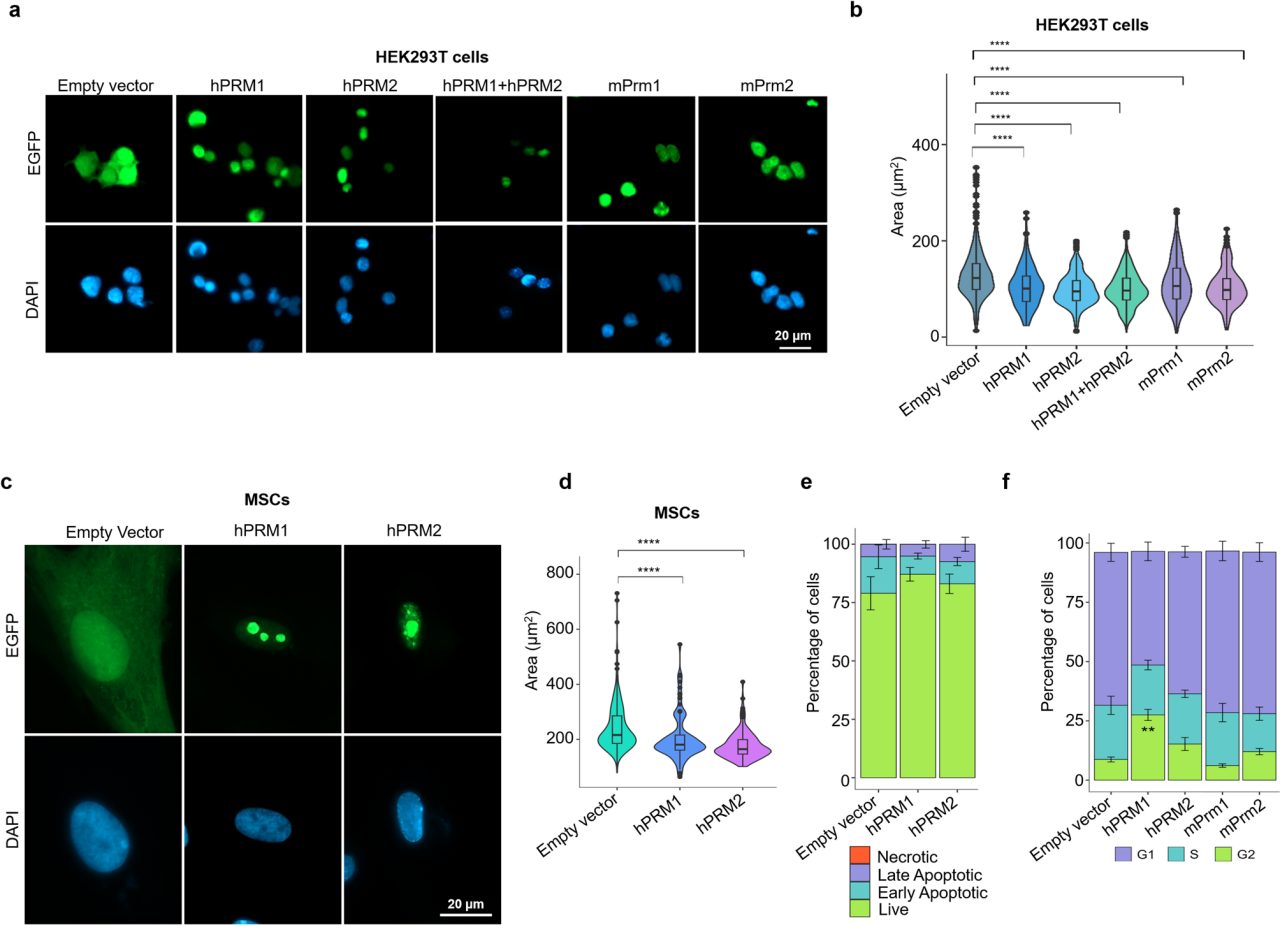
Protamine overexpression leads to nuclear condensation in somatic cells. **(a)** Fluorescence microscopy analysis of HEK293T cells upon overexpression of protamine-EGFP fusion proteins or EGFP-control (nuclei are counterstained with DAPI). **(b)** Nuclear area measurements in transfected cells. Violin plots indicate nuclear area (*n* = 3). P-values were calculated using Welch’s t-test (****: *p* ≤ 0.00001). **(c)** Fluorescence microscopy analysis of control and transfected MSCs counterstained with DAPI. **(d)** Nuclear area measurements of MSCs transfected with empty vector or human protamine plasmids, in analogy to (b). **(e)** Apoptosis analysis of control and transfected HEK293T cells (*n* = 3; error bars indicate the standard deviation). Welch’s t-tests did not reveal significant differences. **(f)** Cell cycle analysis by flow cytometry of control HEK293T and cells transfected with human and murine protamines (*n* = 3; error bars indicate the standard deviation). Significance was estimated with Welch’s t-test adjusted for multiple comparisons using the Benjamini-Hochberg method (**: *p* ≤ 0.001)

HEK293T cells were transfected with plasmids that express human and murine protamines as fusion proteins with enhanced green fluorescent protein (EGFP; Figure S1a, b), and EGFP high-expressing cells were sorted for further analysis. In addition to individual protamine-expressing constructs, HEK293T cells were also transfected with hPRM1and hPRM2 together. The expression of protamines was confirmed at the transcript level (Figure S1c). Whereas localization of EGFP in controls was nuclear as well as cytoplasmic, the human PRM1/PRM2-EGFP fusion proteins and the murine Prm1/Prm2-EGFP fusion proteins exclusively localized to the nucleus (Fig. 1a). The EGFP signal was also detected on mitotic chromosomes of dividing cells, suggesting chromosomal incorporation (Figure S1d). Notably, the nuclear signal was not evenly distributed, but enriched in small nuclear areas that might correspond to nucleoli, especially for hPRM1 (Figure S1b, e and f). Interestingly, while human and mouse PRM2 is cleaved at the N terminus after binding to DNA during spermiogenesis [18], the signal from the N terminal EGFP in hPRM2 transfected cells remains localized to the nucleus after transfection, indicating that in somatic cells, the transfected hPRM2 may not get cleaved. Furthermore, protamine-expressing cells had significantly smaller nuclear areas compared to empty vector-transfected cells, suggesting nuclear condensation. Expression of hPRM1, hPRM2, hPRM1 + hPRM2, mPrm1, and mPrm2 fusion proteins resulted in a reduction of mean nuclear area by 28.88%, 29.62%, 29.31%, 22.22%, and 31.11%, respectively (Fig. 1b). Surprisingly, cotransfection of hPRM1 and hPRM2 did not result in a cumulatively greater reduction in nuclear size. When we transfected human mesenchymal stromal cells (MSCs) with hPRM1 and hPRM2, we observed the same significant reduction in mean nuclear size (22.72% and 34.71%, respectively; Fig. 1c, d). The speckled nucleolar localization, especially for PRM1, was also observed in MSCs (Fig. 1c). Furthermore, similar nuclear changes were observed in mouse embryonic fibroblasts (MEFS; Figure S1f), demonstrating that expression of protamines results in nuclear condensation in somatic cells. Previous studies have shown that GFP-tagged mPRM2 can bind to and condense chromatin in HEK293T cells [18], and our analysis extends these findings to both human and mouse protamines in multiple cell lines. To prevent interspecies bias, and because HEK293T cells exhibited high transfection efficiency, further analysis was limited to HEK293T cells using hPRM1 and hPRM2 constructs.

As nuclear condensation and nuclear membrane destabilization are characteristics of apoptosis [35], we performed Annexin PI staining. No significant differences were observed between the protamine and control conditions when comparing percentages of viable, early apoptotic, late apoptotic, and necrotic cells (Fig. 1e), indicating that the nuclear condensation hardly resulted in apoptosis within 3 days of culture. The effects of protamine expression in HEK293T cells on cell cycle were further characterized by DAPI staining in flow cytometry. We observed a 2.6-fold higher percentage of cells in the G2/M phase and a 1.81-fold lower percentage of cells in the G1 phase in cells expressing hPRM1 compared to empty vector cells. This effect was less in hPRM2-expressing cells and not seen in murine Prm1 or Prm2-expressing cells (Fig. 1f).

### Protamine expression diminishes histone modifications

**Fig. 2:**
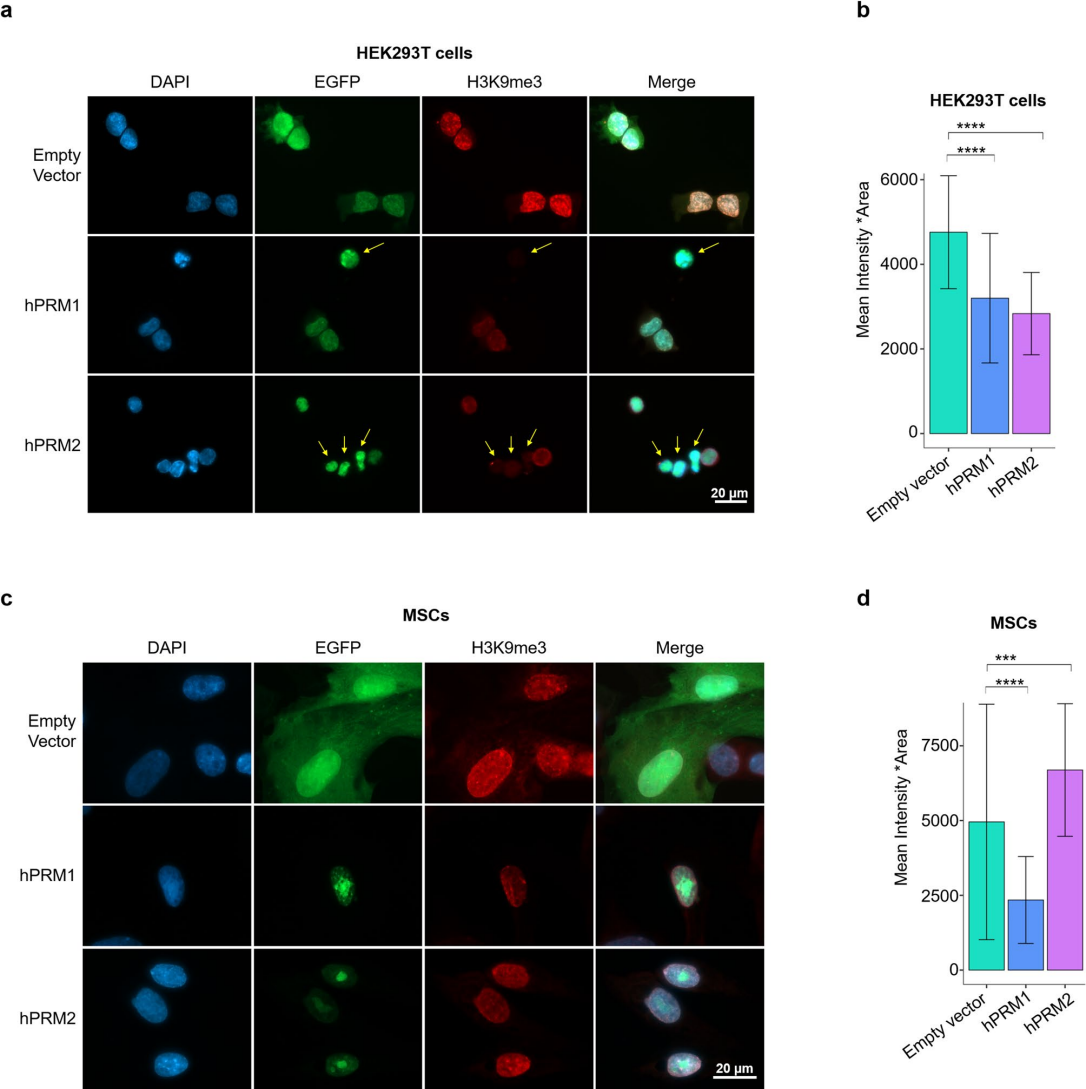
Protamine overexpression leads to displacement of histone modifications. **(a)** Fluorescence microscopy analysis of HEK293T cells transfected with control, hPRM1, or hPRM2 plasmids, and stained for H3K9me3 and DAPI. **(b)** H3K9me3 intensity measurement of HEK293T cells (*n* = 3; mean intensity and standard deviation are indicated). P-values were calculated using Welch’s t-test (****: *p* ≤ 0.00001, ***: *p* ≤ 0.0001). **c**,** d)** Fluorescence microscopy analysis and H3K9me3 intensity measurement in MSCs, in analogy to (a and b)

Sperm protamination is associated with the near-complete eviction of histones during spermiogenesis [7]. To determine whether the nuclear condensation caused by protamine overexpression resulted in a displacement of histones, we quantified histone H3 along with histone modifications. Western blot analysis of global histone levels, H3K9me3, and H3K36me3 did not reveal obvious changes (Figure S2a). However, immunofluorescence staining showed a clear reduction of the H3K9me3 mark in HEK293T cells expressing hPRM1 and hPRM2 (Fig. 2a, b). Overexpression of hPRM1 in MSCs also showed a reduction in histone modification H3K9me3 (Fig. 2c, d). Furthermore, in MSCs, hPRM1 and hPRM2 expression resulted also in a marked reduction of H3K4me1 and H3K27Ac staining (Figure S2b, c). To determine whether the apparent reduction in the histone modification signal is an effect of reduced accessibility of the antibody to the modification because of nuclear condensation, we performed immunofluorescence staining with a pan-histone H3 antibody. H3 staining remained unchanged upon hPRM1 or hPRM2 transfection (Figure S2d). These results indicate that while histones still persist, the nuclear localization of specific histone marks clearly diminishes upon protamine overexpression.

### Protamine expression does not significantly affect DNA methylation

**Fig. 3:**
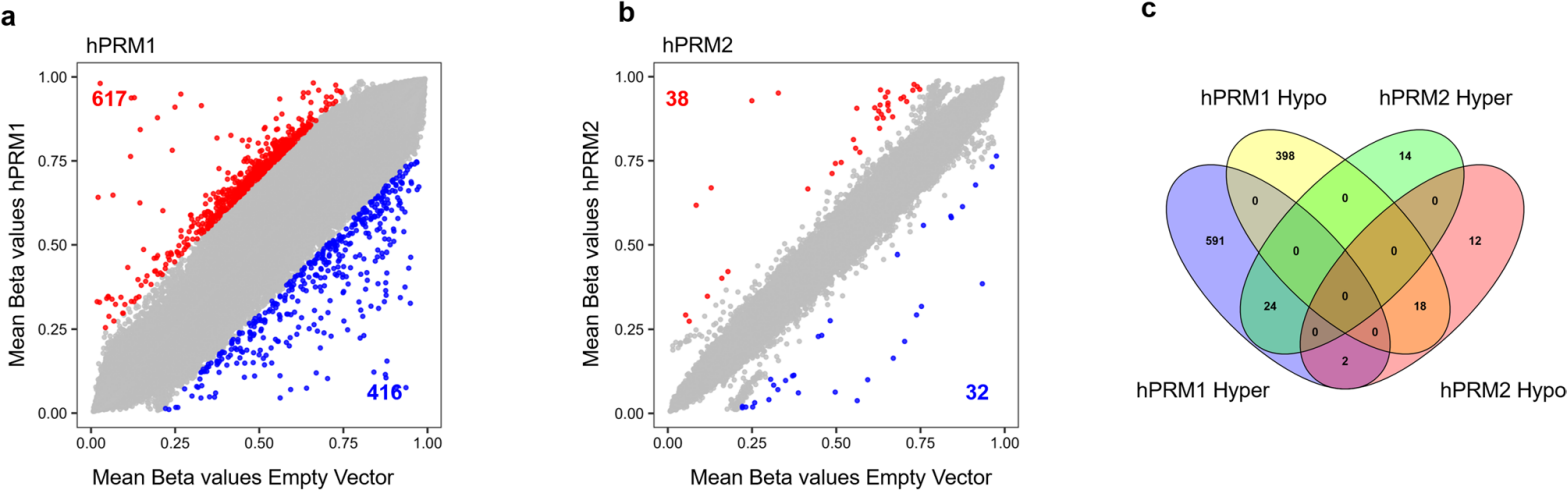
DNA methylome remains stable upon protamine overexpression. **(a-b)** DNA methylation analysis of cells overexpressing hPRM1 (a) or hPRM2 (b). Mean beta values, highlighting hypermethylated (red) and hypomethylated (blue) CpG sites (DiffMean cutoff = 0.2) are depicted, but none of the CpGs reached statistical significance. **c)** Venn diagram depicting overlap of the differentially methylated CpGs upon overexpression of either hPRM1 or hPRM2

The nuclear condensation and partial histone displacement prompted us to investigate whether protamine expression also leads to changes in DNA methylation, as these epigenetic mechanisms are closely related [9, 11]. In fact, DNA methylation changes are very dynamic, occurring already within short timespans, as evidenced by the drastic DNA methylation changes in early development [36]. We therefore performed Illumina BeadChip analysis to determine DNA methylation profiles in controls and protamine-expressing HEK293T cells (*n* = 3). Principal component analysis (PCA) indicated that hPRM1-expressing cells had heterogeneous DNA methylation patterns and clustered a bit apart from the controls and hPRM2-expressing samples (Figure S3a). Notably, despite the clear nuclear condensation and histone modification changes, none of the CG dinucleotides (CpGs) revealed significant differential methylation (limma adjusted P-value < 0.05). For orientation, we focused on the non-significant CpGs that showed at least 20% mean differential methylation. For hPRM1, 617 CpGs were hypermethylated and 416 CpGs were hypomethylated; for hPRM2, 38 CpGs were hypermethylated and 32 were hypomethylated (Fig. 3a, b). There was a moderate overlap between differential DNA methylation of hPRM1 and hPRM2, which might also be due to the same empty vector controls utilized in both experiments (Fig. 3c). Overall, nuclear condensation caused by protamine expression hardly resulted in reproducible changes in DNA methylation.

### Protamine expression severely impairs transcription

**Fig. 4:**
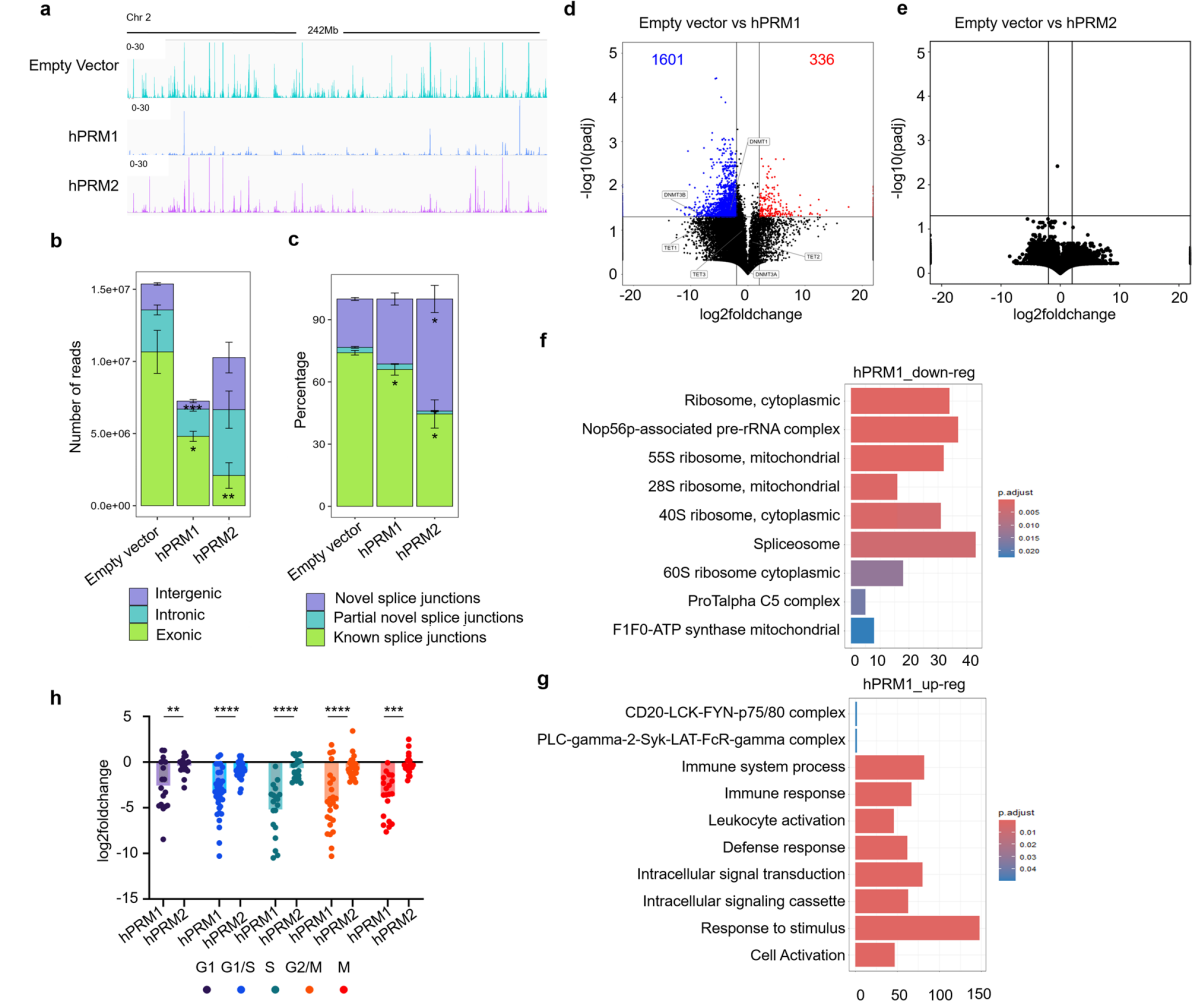
Protamine overexpression leads to severely impaired transcription. **a**) IGV tracks representing normalized transcription in control and transfected cells in a representative genomic region. **(b)** Number and genomic origin of reads obtained by RNAseq in control and hPRM1 and hPRM2 transfected cells classified by Qualimap. (*n* = 3; mean and standard deviation are indicated) **(c)** Splice junctions classified by RSeQC (*n* = 3; mean and standard deviation are indicated). **(d-e)** Volcano plots showing differential gene expression upon overexpression of hPRM1 (**d**) and hPRM2 (**e**) compared to the empty vector controls. Significance thresholds (*p*≤ 0.05 and ≥ 2-fold change) are indicated by the dotted lines. Expression levels of genes coding for DNA-methylating and demethylating proteins are indicated. **(f-g)** Gene ontology analysis of downregulated (f) and upregulated (g) genes in hPRM1-expressing HEK293T cells. The X-axis represents the number of genes in each functional classification. **h)** Gene expression analysis of cell cycle genes in hPRM1 and hPRM2-expressing cells. The Y axis indicates log2fold change compared to the empty vector. Cell cycle genes are divided by the associated cell cycle phase, detailed in Supplemental Table 3. Significance was tested with the Welch’s t-test (**: *p* ≤ 0.001, ***: *p* ≤ 0.0001, ***: *p* ≤ 0.00001).

During spermiogenesis, protamination is associated with an extensive reduction in transcription [37]; however, whether protamines directly cause transcriptional changes in somatic cells has not been explored. We performed RNA-seq analysis of HEK293T cells expressing either hPRM1 or hPRM2, and found overall extensively reduced transcript levels in cells expressing protamines (Fig. 4a). As expected, hPRM1 and hPRM2 were not expressed in non-transfected HEK293T cells. While an average of 23.4 million reads were obtained from control samples, only 10 million and 15.2 million reads were obtained from hPRM1 and hPRM2-expressing samples, respectively. Particularly, exonic reads decreased in hPRM2-expressing cells, while hPRM1-expressing cells exhibited similar percentages compared to empty vector transfection (Fig. 4b). Furthermore, hPRM2-expressing cells exhibited a higher representation of novel splice junctions and a reduction of known splice junctions compared to empty vector and hPRM1 (Fig. 4c). This indicates that while overexpression of either hPRM1 or hPRM2 results in impaired transcription, there may be protamine-specific effects on the transcriptome.

Due to the significantly lower overall level of transcription, the standard pipelines to determine differentially expressed genes (DEGs) may result in a normalization bias [33, 38]. We therefore performed DEG analysis after additional normalization by total number of reads per sample. Interestingly, we were only able to obtain DEGs for hPRM1 overexpression, which resulted in 336 genes upregulated and 1601 genes downregulated, including DNA methyltransferase 1 and 3B (DNMT1 and DNMT3B) and ten-eleven translocation methylcytosine dioxygenase 1 (TET1). In contrast, no significant differential expression was detected in hPRM2-expressing cells. (Fig. 4d, e), which may in part result from the high variability between the replicates of hPRM2-expressing cells (Figure S3b), or a more random binding of hPRM2 to accessible chromatin.

Gene ontology analysis of downregulated genes upon hPRM1 overexpression showed significant enrichment of ribosomal, nucleolar, and splicing-related transcripts, which seem to preferentially include nucleolar transcripts (Fig. 4f). This is in line with previous reports and our own data indicating nucleolar localization of hPRM1 [17]. In contrast, upregulated genes were rather enriched in immune response and signaling pathway-related transcripts (Fig. 4g).

Subsequently, we analyzed differential expression of cell cycle genes (KEGG pathway/ cell cycle/human) in hPRM1 and hPRM2-expressing cells. While modest changes in cell cycle genes were seen upon hPRM2 overexpression, we observed a consistent reduction in cell cycle gene expression in hPRM1-expressing cells. This is particularly observed for S and G2 phase-associated genes (Fig. 4h, Supplemental Table 3), which is consistent with our cell cycle analysis.

## Discussion

Our study demonstrates that overexpression of protamines in somatic cells results in a partial condensation of nuclei with disruption of transcription, while the methylome remains largely unaffected. Furthermore, PRM1 and PRM2 seem to have distinct effects on gene expression.

During spermiogenesis, protamines replace most histones while retaining histones and their modifications at developmentally important genomic locations [8, 39]. Although we did not observe complete histone replacement upon expression of hPRM1 or hPRM2 in HEK293T or MSCs, it should be noted that the incorporation into chromatin and histone replacement during spermiogenesis is a part of a multistep cascade that begins with hyperacetylation of sperm-specific histones, incorporation of histone variants, nucleosome disassembly, DNA binding with transition proteins (TNPs) and finally replacement of the TNPs by protamines, each step sequentially facilitating nuclear compaction and transcription cessation [40]. Iuso et al. generated H2B-GFP cells followed by transfection with Prm1-RFP and observed replacement of GFP by RFP [17]. The discrepancy to the lack of displacement of endogenous H3 in our data could be attributed to differences in cell type and histone tested, given that H2B is more readily displaced compared to H3 [41, 42]. Either way, we could observe a clear loss of H3K9me3 in HEK293T cells and MSCs expressing either hPRM1 or hPRM2. Previous studies demonstrated that transient expression of human or murine protamine 1 in sheep fibroblasts also resulted in loss of H3K9me3 [17, 43]. Furthermore, we saw either a reduction or altered localization of H3K4me1 and H3K27Ac marks upon hPRM1 overexpression in MSCs, which might be attributed to the more open chromatin of MSCs as compared to HEK293T cells [44]. While it cannot be completely ruled out that the nuclear condensation might occlude antibody binding and result in a perceived reduction of histone modifications, pan H3 staining still showed H3 persistence in the nucleus. Previous studies have also demonstrated higher protamination and nuclear condensation in the presence of HDAC inhibitors such as trichostatin (TSA) [17, 43]. Hence, TSA might also enhance nuclear condensation upon protamine expression in somatic cells.

The DNA methylation pattern changes in a highly dynamic manner during cellular development and differentiation [36], and it has been shown that epigenetic crosstalk exists between different regulatory mechanisms [9, 10]. We therefore investigated whether nuclear reorganization with at least partial histone replacement could be reflected in the methylome. However, our results indicate that protamine expression does not evoke significant and directed changes in DNA methylation. In fact, previous studies that compared DNA methylation levels in different stages of mouse spermatogenesis saw a rather stable, highly methylated profile throughout spermatogenesis [45]. Thus, the extensive nuclear condensation upon protamination might render the DNA inaccessible to DNA methylating or demethylating enzymes, resulting in a relatively stable methylome.

It is well known that cessation of transcription occurs during spermiogenesis [1, 16]. However, it is not understood whether and to what extent the protamines directly contribute to this transcriptional stop. Our results demonstrate that hPRM1 and hPRM2 expression severely impaired transcription, with a decline of 57.2% and 35% of the total transcripts, respectively. While the retained sequences showed similar distribution of exonic, intronic and intergenic regions for hPRM1, hPRM2 cells showed a significantly higher retention of intronic and intergenic sequences, which might point to different binding sites of these proteins. In fact, PRM2 is a zinc finger protein with a Cys2His2 motif [46] which may confer a DNA-binding and transcription regulation function distinct from PRM1 [47]. A report by Merges et al. revealed transcriptomic as well as proteomic differences of the testis of Prm1 knockout *versus* Prm2 knockout mice. Interestingly, gene set enrichment analysis showed enrichment of immune response genes in Prm1 -/-, but not Prm2 -/- mice, which is consistent with our results [48]. On the other hand, we observed clear down-regulation of ribosomal RNA genes upon hPRM1 overexpression, and these genes are associated with nucleoli that also showed enriched EGFP signal in fluorescence microscopy. Thus, it appears likely that PRM1 particularly binds to and condenses the nucleolar genomic regions [49] and thereby interferes more with ribosomal genes than the average silencing effect. The binding of protamines in somatic cells in vitro, so far seems to only depend on the electrostatic affinity of the protamines to the DNA, which may be enhanced, for hPRM2 by the presence of zinc [2]. Our results point to a possible directed binding for hPRM1 to nucleoli, and further investigation, especially in the context of the nature of physical interactions of chromatin proteins in the nucleus [50], could shed light on the differences between the affinity and function of the two proteins.

Additionally, the cell cycle defects seen in hPRM1-transfected cells could be correlated with gene expression data that indicated reduction in the expression of cell cycle genes. While further investigation of changes in specific histone modifications would shed light on the mechanisms by which protamination may affect cell cycle, previous reports that suggest that H3K4 methylation and all histone acetylation increase in the S phase [51], are consistent with our observation that H3K27Ac and H3K4me1 decrease in protaminated HEK293T cells and could contribute to the cell cycle defects.

Taken together, protamines can alter chromatin structure and nuclear architecture even in somatic cells. Their property of nucleic acid binding and chromatin condensation lends them to potential applications in nanopharmaceuticals and drug targeting [21, 52]. Protamines are considered to bind to DNA in a sequence-independent manner using electrostatic interactions between the DNA and the arginine-rich protamines [1]. This may preclude genomic regions associated with closed chromatin from being accessible to protamine binding. Our results indicate that hPRM1 and hPRM2 may have different binding preferences. While both proteins lead to nuclear condensation, histone modification changes and severely impaired transcription independent of DNA methylation, particularly the overexpression of hPRM1 resulted in additional significant gene expression changes. It should be noted that the histone to protamine transition in human and mouse relies on a temporally regulated expression of both protamines, with protamine 2 expressed as a precursor, in a specific ratio [40]. While we have not explored this aspect of protamine incorporation in our study, further investigating these differences would not only elucidate the process of sperm protamination but also assist in the development of potential protamine-mediated applications.

## Supplementary Information

Supplementary Material 1.

## Data Availability

The RNA-Seq and DNA methylation data has been submitted on GEO with the access GSE296730 and GSE297024 respectively.
