# Supplementary material for: Protamine expression in somatic cells condenses chromatin and disrupts transcription without altering DNA methylation
Source: Epigenetics Chromatin. 2025 Oct 4;18:64. doi: 10.1186/s13072-025-00633-2 (PMC12495770; doi:10.1186/s13072-025-00633-2)
Supplement: Supplementary file 1 — Supplementary Material 1. [file 13072_2025_633_MOESM1_ESM.docx]

# Supplemental Information

**Protamine expression in somatic cells condenses chromatin and disrupts transcription without altering DNA methylation**

Deepika Puri, Alexandra Bott, Monica Varona Baranda, Esra Dursun Torlak, Gina Esther Merges, Hubert Schorle, Wolfgang Wagner


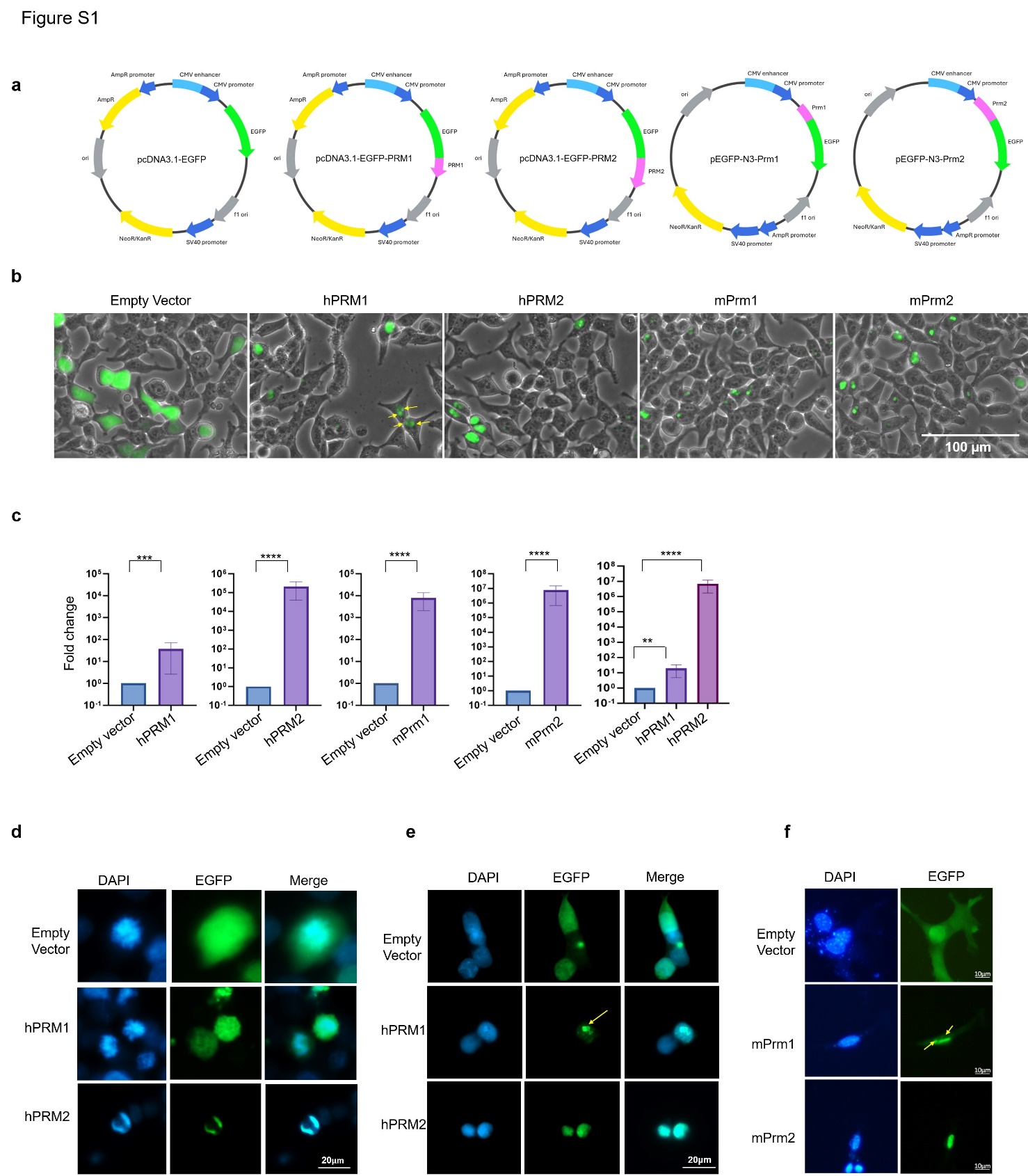
**Figure S1: Protamine overexpression and effects on somatic cells**

**a**) Schemes of plasmids used for the study. **b**) Brightfield images of HEK293T cells transfected with empty vector and human and murine protamines. The speckled localization of hPRM1 is indicated by arrows. **c**) qRT-PCR analysis in HEK293T cells transfected pEGFP-empty vector, hPRM1, hPRM2, mPrm1, mPrm2, and hPRM1+hPRM2 (n = 3; fold change normalized to empty vector samples; error bars indicate standard deviation). P-values were calculated using the Welch’s t-test and adjusted for multiple comparisons using the Benjamini-Hochberg method (**: p ≤ 0.001, ***: p ≤ 0.0001, ****: p ≤ 0.00001). **d**) Immunofluorescence analysis depicting EGFP signal on mitotic chromosomes in HEK293T cells transfected with hPRM1 and hPRM2. The EGFP signal is more diffuse and not confined to the chromosomes in control cells. Nuclei are stained with DAPI. **e**) Immunofuorescence analysis of HEK293T cells transfected with hPRM1 and hPRM2. Cells were imaged at 63x to depict the speckled localization of hPRM1, as highlighted by arrows. **f**) Immunofluorescence analysis of control and transfected MEF cells stained with DAPI. The speckled localization of hPRM1 is depicted by arrows.


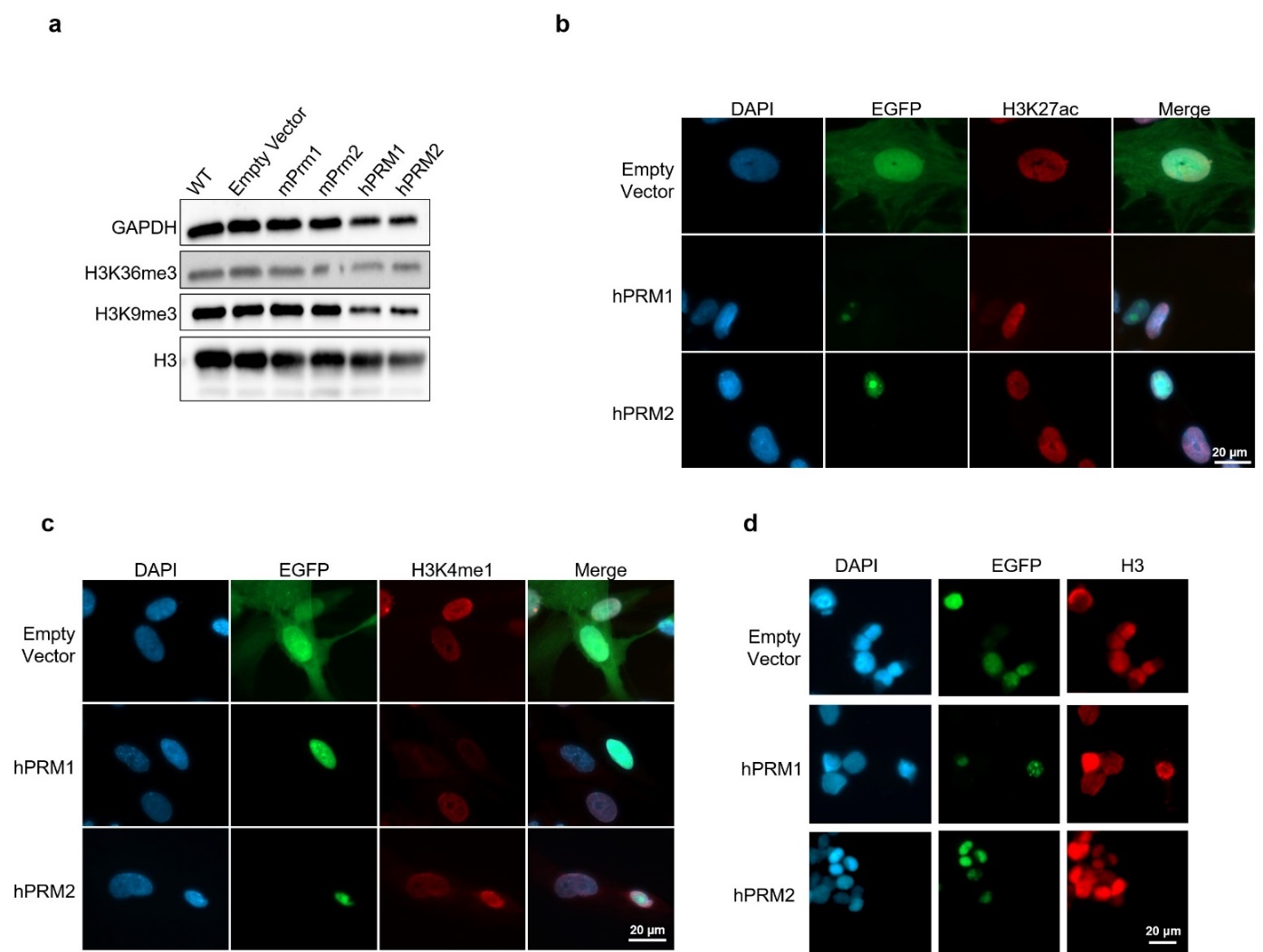


**Figure S2: Protamine overexpression displaces H3K4me1 and H3K27ac**

**a)** Western blot analysis for H3, H3K36me3 and H3K9me3 in control and transfected HEK293T cells. GAPDH is used as a loading control. **b-c**) Immunofluorescence analysis of MSCs transfected with control, hPRM1, or hPRM2 plasmids, stained with antibodies against H3K27ac (b) and H3K4me1 (c) and counterstained with DAPI. **d**) Immunofluorescence analysis of HEK293Tcells transfected with control, hPRM1, or hPRM2 plasmids, stained with an antibody against H3 and counterstained with DAPI.


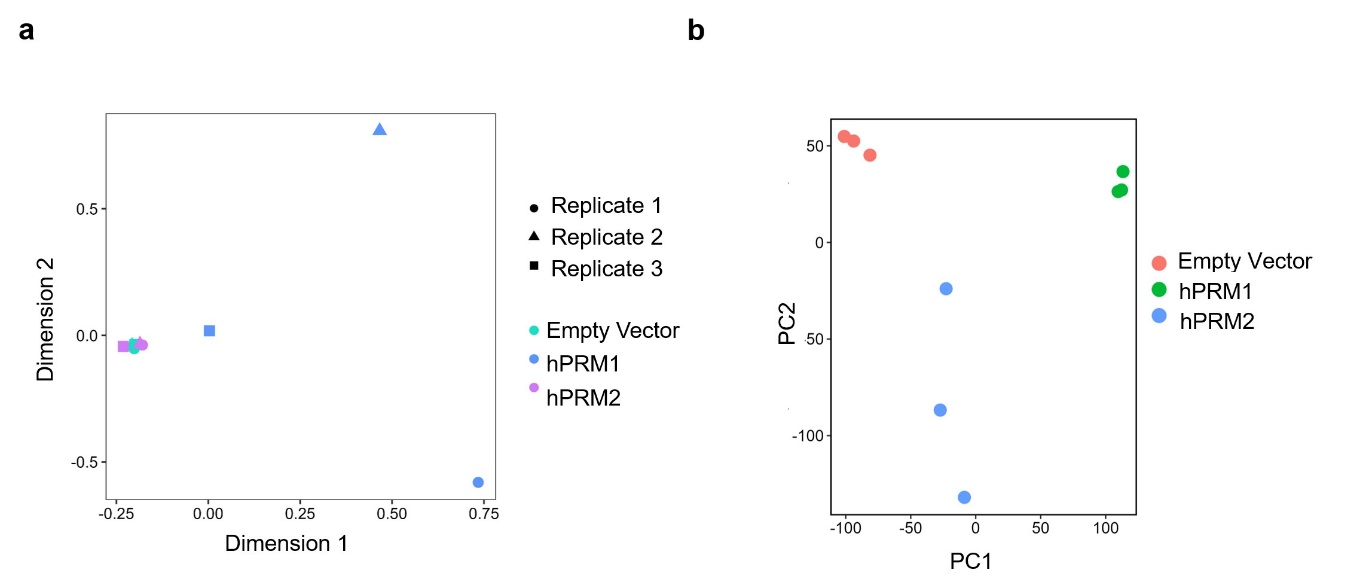
**Figure S3: Sample distribution of DNA methylation and RNA-sequencing**

a) MDS analysis of the top 10,000 differentially methylated regions in control, hPRM1 and hPRM2-transfected cells. Individual replicates are indicated. b) PC analysis of RNAseq from control, hPRM1 and hPRM2-transfected cells

**Table S1: Primers used for qRT-PCR**

| **Target gene** | **Primer Name** | **Direction** | **Sequence** |
| --- | --- | --- | --- |
| GAPDH | GAPDH F | Forward | GAAGGTGAAGGTCGGAGTC |
| GAPDH | GAPDH R | Reverse | GAAGATGGTGATGGGATTTC |
| PRM1 (human) | hPRM1f1 | Forward | ATCCACCAAACTCCTGCCTG |
| PRM1 (human) | hPRM1r1 | Reverse | ACAGGCGGCATTGTTCCTTA |
| PRM2 (human) | hPRM2f1 | Forward | CGTCGAGGTCTACGAGAGGA |
| PRM2 (human) | hPRM2r1 | Reverse | CCTGGTTCTGCAGCCTCTG |
| Prm1 (mouse) | mPrm1f1 | Forward | CCGCCGCTCATACACCATAA |
| Prm1 (mouse) | mPrm1r1 | Reverse | GTTTTTCATCGGACGGTGGC |
| Prm2 (mouse) | mPrm2f4 | Forward | TATGGGAGGACACACAGGGG |
| Prm2 (mouse) | mPrm2r4 | Reverse | CCTCCTTCGGGATCTTCTGC |

**Table S2: Antibodies used in this study**

| **Target** | **Host Species** | **Manufacturer** | **Catalog Number** | **Concentration for immunostaining** | **Concentration for Western blot** |
| --- | --- | --- | --- | --- | --- |
| **Primary Antibodies** | |  |  |  |  |
| H3K4me1 | Rabbit | Abcam | ab8895 | 0.5 µg/ml | 1:3000 |
| H3K9me3 | Rabbit | Abcam | ab8898 | 0.5 µg/ml | 1:2500 |
| H3K27ac | Rabbit | Abcam | ab4729 | 1 µg/ml | 1:3000 |
| H3 | Rabbit | Abcam | ab1791 | 0.5 µg/ml | 1:3000 |
| **Secondary Antibody** | |  |  |  |  |
| Goat anti Rabbit Alexa Fluor 594 | | Invitrogen | A11012 | 2 µg/ml |  |

**Table S3: Differential expression of cell cycle genes upon transfection with hPRM1 or hPRM2**

| **ENSEMBL ID** | **Gene** | **Cell cycle phase** | **Log fold change hPRM1** | **Log fold change hPRM2** |
| --- | --- | --- | --- | --- |
| ENSG00000110092 | *CCND1* | G1 | -6.483798591 | 0.028537 |
| ENSG00000118971 | *CCND2* | G1 | -1.767246014 | -0.09088 |
| ENSG00000112576 | *CCND3* | G1 | 1.266676933 | -0.95577 |
| ENSG00000123374 | *CDK2* | G1 | -4.882459536 | 0.186106 |
| ENSG00000135446 | *CDK4* | G1 | -5.085008638 | -0.12577 |
| ENSG00000129757 | *CDKN1C* | G1 | 0.524635736 | 1.016978 |
| ENSG00000147889 | *CDKN2A* | G1 | -8.463171279 | 0.600984 |
| ENSG00000166508 | *MCM7* | G1 | -4.754072631 | -0.24377 |
| ENSG00000103479 | *RBL2* | G1 | 1.291804303 | -0.81024 |
| ENSG00000077935 | *SMC1B* | G1 | -4.785890058 | 0 |
| ENSG00000166913 | *YWHAB* | G1 | -0.64627026 | -0.74849 |
| ENSG00000108953 | *YWHAE* | G1 | -4.644534539 | 0.699791 |
| ENSG00000170027 | *YWHAG* | G1 | -3.312114285 | -0.85642 |
| ENSG00000128245 | *YWHAH* | G1 | -2.84204992 | -1.93966 |
| ENSG00000134308 | *YWHAQ* | G1 | -3.683900199 | -0.18183 |
| ENSG00000164924 | *YWHAZ* | G1 | -1.88631151 | -0.44802 |
| ENSG00000116809 | *ZBTB17* | G1 | -1.888216735 | -2.80453 |
| ENSG00000097007 | *ABL1* | G1-S | -3.756505274 | -1.05877 |
| ENSG00000105173 | *CCNE1* | G1-S | -5.677796952 | -0.94193 |
| ENSG00000175305 | *CCNE2* | G1-S | -5.582602227 | -0.86637 |
| ENSG00000170312 | *CDK1* | G1-S | -10.28390676 | -2.95086 |
| ENSG00000134058 | *CDK7* | G1-S | -3.170036422 | -1.64644 |
| ENSG00000124762 | *CDKN1A* | G1-S | -2.614628902 | 0.681096 |
| ENSG00000111276 | *CDKN1B* | G1-S | -0.195360988 | -1.08602 |
| ENSG00000147883 | *CDKN2B* | G1-S | -4.444488932 | 0.680148 |
| ENSG00000123080 | *CDKN2C* | G1-S | -5.728654316 | 0.360457 |
| ENSG00000005339 | *CREBBP* | G1-S | 0.778815438 | -0.18395 |
| ENSG00000006634 | *DBF4* | G1-S | -4.853194807 | -0.77124 |
| ENSG00000101412 | *E2F1* | G1-S | -6.385827899 | -1.91718 |
| ENSG00000007968 | *E2F2* | G1-S | -0.188471052 | -1.42941 |
| ENSG00000112242 | *E2F3* | G1-S | -1.145806575 | -0.15205 |
| ENSG00000205250 | *E2F4* | G1-S | -1.106208256 | -0.94227 |
| ENSG00000130222 | *GADD45G* | G1-S | -0.564809515 | -0.98099 |
| ENSG00000082701 | *GSK3B* | G1-S | -2.09357493 | -3.33291 |
| ENSG00000164109 | *MAD2L1* | G1-S | -8.857785007 | -0.6113 |
| ENSG00000116670 | *MAD2L2* | G1-S | -3.415541293 | -0.77377 |
| ENSG00000112118 | *MCM3* | G1-S | -4.103286367 | -0.97667 |
| ENSG00000100297 | *MCM5* | G1-S | -3.906071628 | -1.37421 |
| ENSG00000135679 | *MDM2* | G1-S | -2.393068962 | -0.06977 |
| ENSG00000115947 | *ORC4* | G1-S | -3.121646949 | -1.4743 |
| ENSG00000164611 | *PTTG1* | G1-S | -7.15659455 | -0.87338 |
| ENSG00000250254 | *PTTG2* | G1-S | 0 | 0 |
| ENSG00000164754 | *RAD21* | G1-S | -2.011059002 | -1.01571 |
| ENSG00000139687 | *RB1* | G1-S | -2.731553023 | -0.6122 |
| ENSG00000080839 | *RBL1* | G1-S | -3.252445837 | -1.35896 |
| ENSG00000100387 | *RBX1* | G1-S | -5.029089283 | 0.007037 |
| ENSG00000175793 | *SFN* | G1-S | -0.182358416 | -1.74212 |
| ENSG00000113558 | *SKP1* | G1-S | -2.389091448 | 0.031356 |
| ENSG00000072501 | *SMC1A* | G1-S | -3.382129588 | -0.83293 |
| ENSG00000108055 | *SMC3* | G1-S | -4.045505063 | -0.83354 |
| ENSG00000118007 | *STAG1* | G1-S | 0.590003527 | -0.16223 |
| ENSG00000101972 | *STAG2* | G1-S | -2.940036287 | -0.67759 |
| ENSG00000133101 | *CCNA1* | S | 0 | 0.884451 |
| ENSG00000145386 | *CCNA2* | S | -10.49322011 | -0.36469 |
| ENSG00000134480 | *CCNH* | S | -4.318662399 | -2.07516 |
| ENSG00000004897 | *CDC27* | S | -4.05572988 | -1.46754 |
| ENSG00000093009 | *CDC45* | S | -9.725452259 | 0.789975 |
| ENSG00000094804 | *CDC6* | S | -10.20393038 | 0.887109 |
| ENSG00000105810 | *CDK6* | S | -2.472439283 | -1.8209 |
| ENSG00000055130 | *CUL1* | S | -3.522514828 | -1.09849 |
| ENSG00000133740 | *E2F5* | S | -5.285494548 | -2.29107 |
| ENSG00000105325 | *FZR1* | S | -2.898763532 | 0.099585 |
| ENSG00000116717 | *GADD45A* | S | -3.799981611 | -1.18359 |
| ENSG00000099860 | *GADD45B* | S | -0.47387448 | 0.158261 |
| ENSG00000073111 | *MCM2* | S | -6.845878367 | -1.09881 |
| ENSG00000104738 | *MCM4* | S | 0 | 0.892191 |
| ENSG00000076003 | *MCM6* | S | -7.154704029 | -1.76843 |
| ENSG00000136997 | *MYC* | S | -3.625870969 | -0.05552 |
| ENSG00000085840 | *ORC1* | S | -8.327417028 | -0.95127 |
| ENSG00000115942 | *ORC2* | S | -1.935755135 | -1.45149 |
| ENSG00000135336 | *ORC3* | S | -5.314511131 | -0.26137 |
| ENSG00000164815 | *ORC5* | S | -4.703596018 | -2.24895 |
| ENSG00000198176 | *TFDP1* | S | -3.560234121 | 0.695074 |
| ENSG00000149311 | *ATM* | G2 | 1.032257571 | 1.196678 |
| ENSG00000175054 | *ATR* | G2 | -3.973239569 | -0.79739 |
| ENSG00000169679 | *BUB1* | G2 | 0 | -1.64445 |
| ENSG00000156970 | *BUB1B* | G2 | -7.659408531 | -0.69712 |
| ENSG00000154473 | *BUB3* | G2 | -3.845764255 | -0.19756 |
| ENSG00000134057 | *CCNB1* | G2 | -6.872581256 | -0.07898 |
| ENSG00000157456 | *CCNB2* | G2 | -9.444169454 | -1.22104 |
| ENSG00000147082 | *CCNB3* | G2 | 0 | 3.405339 |
| ENSG00000094880 | *CDC23* | G2 | -4.456878573 | -0.59904 |
| ENSG00000164045 | *CDC25A* | G2 | -10.30725416 | -1.24913 |
| ENSG00000101224 | *CDC25B* | G2 | 0.351024309 | -0.5335 |
| ENSG00000097046 | *CDC7* | G2 | -6.363690407 | -1.29946 |
| ENSG00000129355 | *CDKN2D* | G2 | 1.886195673 | -2.2035 |
| ENSG00000149554 | *CHEK1* | G2 | -7.916702202 | -0.57085 |
| ENSG00000183765 | *CHEK2* | G2 | -5.278985695 | -1.33617 |
| ENSG00000116478 | *HDAC1* | G2 | -3.00023089 | -0.35671 |
| ENSG00000196591 | *HDAC2* | G2 | -4.679956037 | -0.10493 |
| ENSG00000002822 | *MAD1L1* | G2 | -1.436178975 | -1.83993 |
| ENSG00000091651 | *ORC6* | G2 | -6.161177175 | -0.80704 |
| ENSG00000132646 | *PCNA* | G2 | -6.008436429 | -1.19834 |
| ENSG00000127564 | *PKMYT1* | G2 | -7.86901391 | -1.89613 |
| ENSG00000114126 | *TFDP2* | G2 | -4.008409545 | -2.17156 |
| ENSG00000105329 | *TGFB1* | G2 | 1.12675938 | -1.08318 |
| ENSG00000092969 | *TGFB2* | G2 | -4.345571587 | 0.676657 |
| ENSG00000119699 | *TGFB3* | G2 | -2.28467127 | 0.205768 |
| ENSG00000141510 | *TP53* | G2 | -1.85561441 | -0.2028 |
| ENSG00000112742 | *TTK* | G2 | 0 | -0.82526 |
| ENSG00000166483 | *WEE1* | G2 | -4.899362869 | 0.282959 |
| ENSG00000214102 | *WEE2* | G2 | 0 | 0 |
| ENSG00000153107 | *ANAPC1* | M | -3.526878224 | 2.48796 |
| ENSG00000164162 | *ANAPC10* | M | -6.91439807 | -0.67022 |
| ENSG00000141552 | *ANAPC11* | M | -3.473026395 | 0.140831 |
| ENSG00000129055 | *ANAPC13* | M | -3.61045587 | 0.294133 |
| ENSG00000176248 | *ANAPC2* | M | -2.884903357 | 0.167562 |
| ENSG00000053900 | *ANAPC4* | M | -1.713734333 | 0.689746 |
| ENSG00000089053 | *ANAPC5* | M | -2.310041992 | -0.3413 |
| ENSG00000196510 | *ANAPC7* | M | -2.541569327 | -0.33024 |
| ENSG00000081377 | *CDC14B* | M | -4.251645401 | 1.753676 |
| ENSG00000130177 | *CDC16* | M | -1.4209012 | -1.53716 |
| ENSG00000117399 | *CDC20* | M | -6.785486823 | -0.4334 |
| ENSG00000158402 | *CDC25C* | M | 0 | -1.5255 |
| ENSG00000176386 | *CDC26* | M | -3.598024824 | -0.52177 |
| ENSG00000100393 | *EP300* | M | -0.06985722 | 0.970362 |
| ENSG00000135476 | *ESPL1* | M | -7.648433942 | -0.61631 |
| ENSG00000166851 | *PLK1* | M | -7.103665716 | -0.58327 |
| ENSG00000253729 | *PRKDC* | M | -5.923904653 | -0.31832 |
| ENSG00000145604 | *SKP2* | M | -6.505090876 | -0.93148 |
| ENSG00000175387 | *SMAD2* | M | -2.073333893 | -0.7169 |
| ENSG00000166949 | *SMAD3* | M | -0.985881795 | -1.0807 |
| ENSG00000141646 | *SMAD4* | M | -1.375626935 | -2.0312 |
